# The Missing Piece in Biosynthesis of Amphidinols: First Evidence of Glycolate as a Starter Unit in New Polyketides from *Amphidinium carterae*

**DOI:** 10.3390/md15060157

**Published:** 2017-05-31

**Authors:** Adele Cutignano, Genoveffa Nuzzo, Angela Sardo, Angelo Fontana

**Affiliations:** Institute of Biomolecular Chemistry, National Research Council (CNR), Pozzuoli 80078, Naples, Italy; nuzzo.genoveffa@icb.cnr.it (G.N.); angela.sardo@icb.cnr.it (A.S.); angelo.fontana@icb.cnr.it (A.F.)

**Keywords:** amphidinol, amphidinol-related polyketide, *Amphidinium*, dinoflagellate, glycolate, polyketide biosynthesis, PKS, antifungal polyketide, *Candida albicans*, SHAM

## Abstract

Two new members of the amphidinol family, amphidinol A (**1**) and its 7-sulfate derivative amphidinol B (**2**), were isolated from a strain of *Amphidinium carterae* of Lake Fusaro, near Naples (Italy), and chemically identified by spectroscopic and spectrometric methods. Amphidinol A showed antifungal activity against *Candida albicans* (MIC = 19 µg/mL). Biosynthetic experiments with stable isotope-labelled acetate allowed defining the elongation process in **1**. For the first time the use of glycolate as a starter unit in the polyketide biosynthesis of amphidinol metabolites was unambiguously demonstrated.

## 1. Introduction

Marine microorganisms are an impressive source of secondary metabolites with complex chemical structures and remarkable biological properties [1]. In our ongoing search for novel marine bioactive molecules [2], we recently focused our attention on microorganisms from the Mediterranean region. In particular, from Lake Fusaro, near Naples (Italy), we isolated a dinoflagellate strain, which was taxonomically identified as *Amphidinium carterae*. The methanol extract of the new isolate displayed antifungal activity against *Candida albicans* (MIC = 64 µg/mL) and its preliminary chemical analysis revealed the occurrence of polyoxygenated polyketide compounds related to amphidinols, a family of long-chain polyhydroxy polyketides including polyene-amphidinols and other related metabolites [3,4,5,6,7,8,9,10,11,12,13,14,15,16,17,18,19,20,21,22,23]. They have been mostly reported from dinoflagellates of the genus *Amphidinium* and exhibited potent biological activities, including antifungal, cytotoxic, and hemolytic properties. From a chemical point of view, all of the members of the family contain a core of two tetrahydropyrans linked by a C6 alkyl chain. One of the most intriguing aspects of these natural products is the biosynthetic assembly of the polyketide chain. Studies reported so far clarify the diverse and peculiar acetate incorporation pattern, leaving unsolved the question about the starter unit, not labelled by this precursor. In analogy with biosynthesis of okadaic acid and congeners [24] glycolate was suggested as a candidate precursor, but no evidence has been provided to date. Here we describe the structural elucidation, biological activity, and the results of biosynthetic studies on two amphidinols from the newly-isolated strain of *A. carterae.*

## 2. Results and Discussion

**Figure 1:**
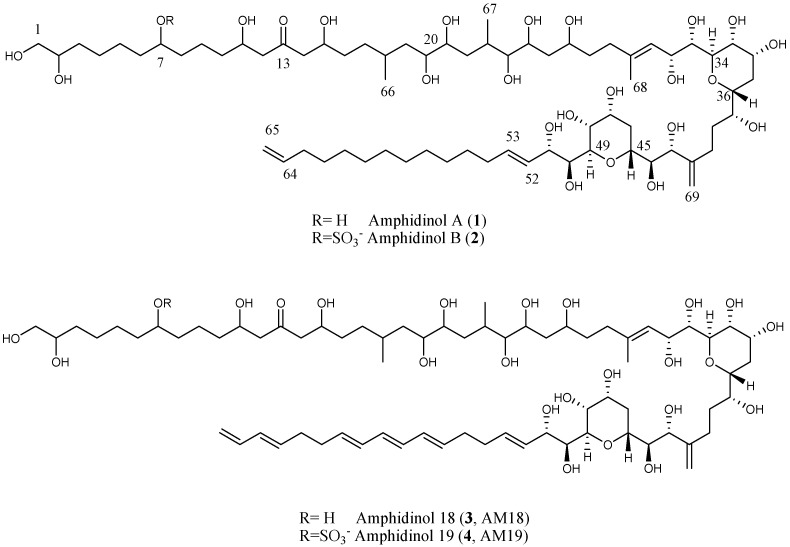
Structures of amphidinols **1**–**4**.

The marine microalgal strain was isolated from seaweed samples collected at Lake Fusaro, near Naples (Italy) in June 2012. The culture was grown in 8 × 1.8 L Fernbach bottles for two weeks. The cell pellet was extracted with methanol and the resulting organic extract was fractionated by a Chromabond Hydra C-18 cartridge using a MeOH/H_2_O gradient according to our reported procedure [21]. Further purification by reversed phase HPLC gave pure compounds **1** (1.6 mg) and **2** (1.0 mg) (Figure 1). HRESIMS of the molecular ion (sodium adduct) of **1** at *m*/*z* 1361.8537 accounted for the molecular formula C_69_H_126_O_24_. A comparison of ^1^H NMR of polyketide **1** with AM18 (**3**) which we previously reported from a commercial strain of *A. carterae* [21], suggested strict structural analogies (Figure 1). Most of the signals were superimposable, with the main differences emerging only in the olefinic region of the spectrum due to the absence of conjugated double bonds in compound **1**.

**Figure 2:**
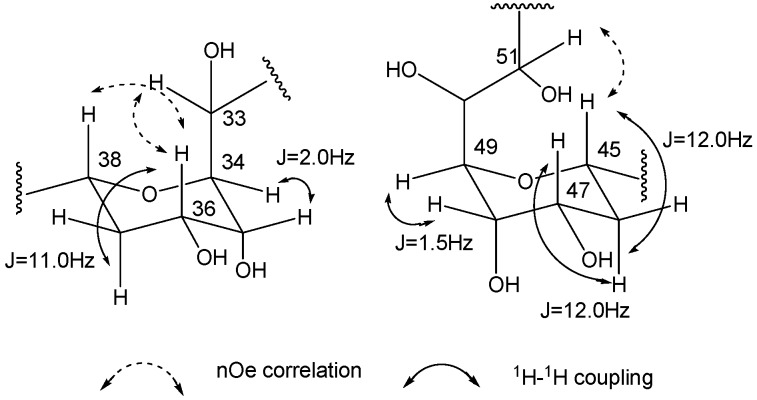
Relative configurations of the tetrahydropyran rings of amphidinol A (**1**).

A careful inspection of mono- (^13^C), homo-, and hetero-nuclear-bidimensional NMR data (Table 1 and Supplementary Materials) allowed identifying in the molecular skeleton of the isolated metabolite **1** the same substructure C1–C53 of **3**, including the 7-hydroxyl group, the β,β’-dihydroxyketone functionality at C13 and the typical *bis*-tetrahydropyrans core. These latter rings showed the same stereostructure described for **3**, deduced by J couplings and nOe effects analysis, as depicted in Figure 2.

The structural similarities with AM18 (**3**) were confirmed by interpretation of the ESI-MS/MS spectrum of **1** registered on the molecular ion [M+Na]^+^ that contained the common fragments at *m*/*z* 669.3810, 687.3916, 745.4333, and 963.5837, as well as the fragment at *m*/*z* 169.0466, diagnostic for the assignment of the isolated hydroxyl function at position 7 (Figure S21). From the other side, a saturated alkyl chain (C54–C63) joined the olefinic carbon C53 (135.3 ppm; H53, δ 5.85) to the terminal double bond (C64, 140.1 ppm; H64, δ 5.77 and C65, 115.0 ppm; H65, δ 4.91, 4.97). According to these observations, **1** differed from **3** in the structure of the aliphatic chain (C52–C65) which was shorter by two carbon atoms and did not contain the conjugated triene moiety in agreement with the seven degrees of formal unsaturations suggested by the molecular formula. The polyketide **1** represented a new member of the amphidinol family and, thus, is named amphidinol A (**1**). A terminal saturated alkyl chain is rather uncommon within this group of polyketides and has been so far described in few analogues isolated from *Amphidinium *sp., such as lingushiol [13], and karatungiols A and B [16]. Like congener AM18, amphidinol A inhibited the growth of *C. albicans* at 19 µg/mL (MIC), thus accounting for the antifungal properties of the extract.

Along with the microalgal metabolite **1**, HPLC purification afforded its 7-sulfate derivative **2** (Figure 1). This latter metabolite was typically recognizable by both chemical shifts of the deshielded oxymethine signals at C7 (80.3 ppm, δ 4.39) and the diagnostic sulfate product ions at *m*/*z* 96.9589 (HSO_4_^−^) in MS/MS spectrum of its molecular ion at *m*/*z* 1417.8187 [M-H]^−^ (Figure S22) and at 142.9386 (HSO_4_Na_2_^+^) in the MS/MS spectrum of the sodium adduct [M-H+2Na]^+^ at *m*/*z* 1463.7912 (Figure S23). The sulfate ester **2** has never been reported before, and was then named amphidinol B. Compound **2** did not show activity against *C. albicans* when assayed up to 150 µg/mL, thus confirming, as observed for AM19 (**4**), that sulfate substitution affects antifungal activity on these molecular skeletons [21].

Biosynthesis of marine polyketides from dinoflagellates is a stimulating field of study that has been hampered, so far, by a still-limited knowledge on the biochemistry and molecular genetics of these metabolites [25]. Furthermore, technical obstacles arose from intrinsic factors such as the slow growth rate of dinoflagellates, low levels of polyketide metabolites, and the scrambling of labelled carbons of precursors. In this scenario, very few studies have been reported in the literature addressing the biosynthesis of dinoflagellate polyketides, mostly focused on polyether toxins and macrolides [25], and only two research groups have performed studies on amphidinols by stable isotope feeding experiments [6,11]. According to this literature, the regular elongation by acetate units, as expected for a polyketide synthase (PKS) catalyzed process, is interrupted in a few sites by a Favorskii-type rearrangement, which involves C1 deletions and an atypical labelling pattern from the acetate precursor. This chemistry, although unusual, has been also reported in polyketides of bacteria [26,27], fungi [28], and in other dinoflagellates (i.e., in amphidinolides and polyether toxins) [25,29]. However, the identity of the biosynthetic starter unit, which was not labelled by acetate, was proposed as glycolate in amphidinols in analogy with other microalgal polyketides [23]. In order to shed light on this point and complete the picture of the biosynthetic assembly for this class of metabolites, we run a set of feeding experiments with labelled precursors, i.e., [1-^13^C]-acetate, [2-^13^C]-acetate, [1,2-^13^C_2_]-acetate, and [1-^13^C]-glycolate to detect by ^13^C NMR the carbon enrichments in the molecular skeleton of **1**, the most abundant polyketide.

**Figure 3:**
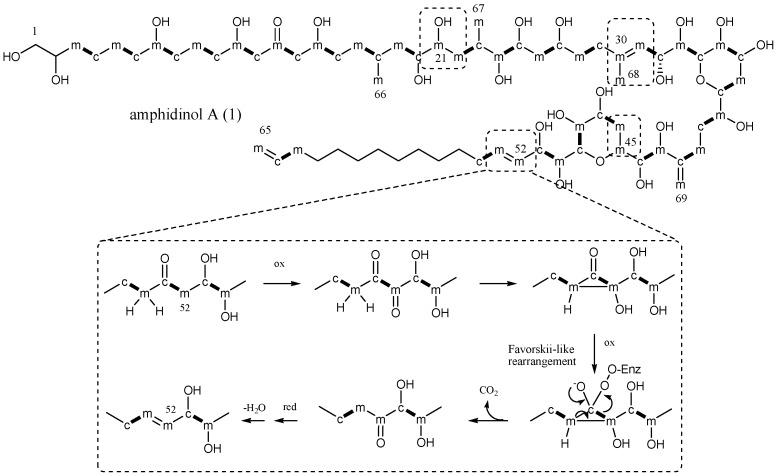
Acetate labelling pattern of amphidinol A (**1**). c: carbons labelled from [1-^13^C]-acetate. m: carbons labelled from [2-^13^C] acetate. c-m: incorporation of intact acetate unit from [1,2-^13^C_2_]-acetate. Discontinuous labelling is evidenced with the dashed boxes. The inset shows a proposed mechanism for a Favorskii-type rearrangement from [26].

Carbon labelling by feeding experiments with ^13^C-acetate precursors, as depicted in Figure 3 revealed a basically regular chain elongation in the frame C3/C20. In fact, a single rearrangement was evidenced by the labelling of the methyl group at 21.3 ppm (C66), resultantly enriched with [2-^13^C]-acetate. Labelling from C21 to C53 was identical to that reported for AM4, which indeed shares the same substructure [6]. Thus, four sites of discontinuity were detected for the positions C21/C22, C30/C31/C68, C45/C46, and C52/C53, which were all labelled by [2-^13^C]-acetate, along with the pendant methyl group at 14.2 ppm (C67) and the olefinic methylene at 113.0 ppm (C69). Carbon signals of the terminal saturated arm C54–C63 could not be unambiguously assigned due to severe overlapping. However, five aliphatic signals in the segment C54–C63 resultantly increased by [1-^13^C]-acetate at 29.9, 30.2, 30.5, 30.7, and 34.6 ppm, and five from [2-^13^C]-acetate at 30.1, 30.3, 30.55, 30.8, and 34.6 ppm. The experiment with doubly-labelled acetate confirmed a regular pattern due to intact C_2_-unit incorporation in this part, showing all of the aliphatic signals resonating as doublets flanking the natural isotopomer carbons (Figure S17). Furthermore, due to skeleton rearrangement and cleavage of the original C_2_ unit, signals at 14.2 (C67), 17.4 (C68), 21.3 (C66), 70.6 (C45), 72.6 (C21), 113.0 (C69), and 129.1 (C52) ppm resonated as singlets. Additional information was inferred about the terminal double bond, whose signal at 140.1ppm (C64) resonated as a doublet (*J* = 22 Hz) while methylene at 115.0 ppm (C65) appeared as a singlet signal (Figure S17), possibly due to a terminal decarboxylation step. As expected, no labelling was observed for positions 1 and 2 of the polyketide chain.

**Figure 4:**
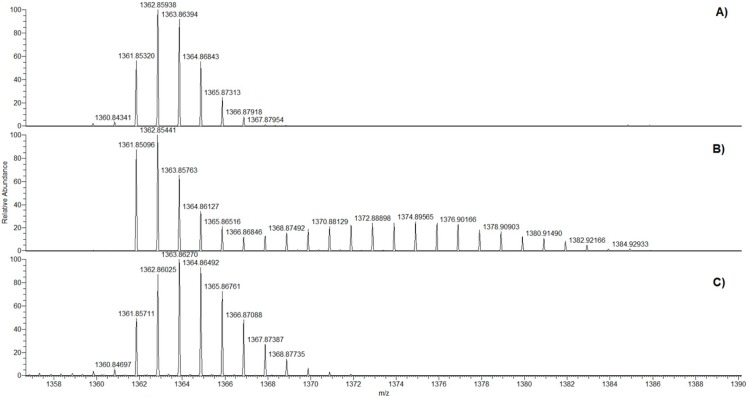
HRESI^+^-MS spectra of amphidinol A (**1**). (**A**) Natural abundance profile; (**B**) labelling after the [1-^13^C]-glycolate feeding experiment; and (**C**) labelling after the [1-^13^C]-glycolate/SHAM feeding experiment.

On the other hand, HRESIMS analysis of **1** after the feeding experiment with [1-^13^C]-glycolate revealed an isotopic cluster centered around *m*/*z* 1375 indicating a random incorporation of several labelled atoms in the molecule (Figure 4B).

As a confirmation, the ^13^C NMR spectrum of **1** showed a general enrichment of every carbon together with the presence of flanking doublets for the statistically occurrence of two vicinal ^13^C in the same skeleton (Figure S18). This multiple labelling was interpreted as the result of an oxidative pathway occurring on the glycolate substrate with the metabolic release via a glyoxylate intermediate of CO_2_, which may be recycled by microalgal photosynthetic processes in a very efficient way.

**Figure 5:**
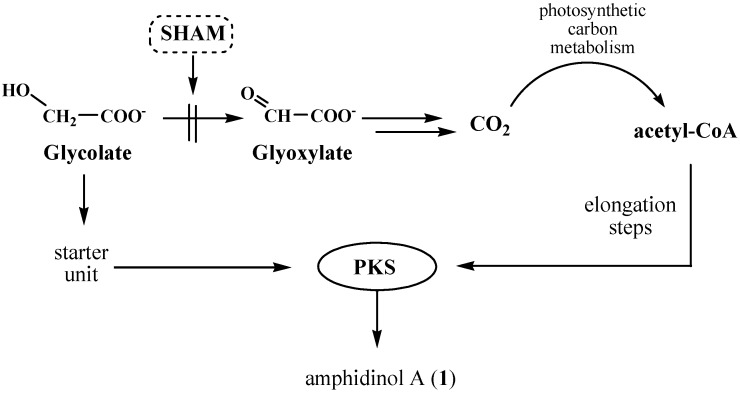
Proposed route of glycolate degradation via glyoxylate and cross-talking with biosynthesis of amphidinol A (**1**) in *A. carterae*. Inhibition of the oxidative pathway by SHAM stops recycling of glycolate carbon by photosynthetic metabolism, but does not affect polyketide biosynthesis by PKS.

Glycolate is a product of photorespiration, one of the major carbon metabolism pathways in oxygen-producing photosynthetic organisms. This process, started by an oxygenase reaction of Rubisco, competes with photosynthetic CO_2_ fixation, causing a loss of carbon, nitrogen, and energy. Thus, a series of strategies have been developed by photosynthetic organisms to efficiently recycle glycolate and other undesired products of photorespiration. In higher plants and multicellular algae, glycolate is oxidized to glyoxylate by a glycolate oxidase in the peroxisomes [30]. In turn, glyoxylate is metabolized to glycine that, in mitochondria, by conversion into serine, releases CO_2_ and NH_4_^+^. Instead, in unicellular green algae, it has been described a major light-dependent glycolate oxidizing system mainly located in the chloroplasts and associated with the photosynthetic electron transport apparatus [31]. In these microorganisms glyoxylate is degraded to CO_2_, which can be recycled and fixed by photosynthetic machinery. The glycolate-oxidizing enzyme is specifically associated with a quinone oxidoreductase system and is inhibited by salicylhydroxamic acid (SHAM) without affecting CO_2_ fixation in intact cells [32]. Little is known on the glycolate metabolism in dinoflagellates, thus, our working hypothesis was that a similar putative oxido-reductase may be involved in glycolate oxidation in microalgal *Amphidinium* cells. Degradation of [1-^13^C]-glycolate in *A. carterae* may afford labelled CO_2_ which is incorporated in primary metabolites, whose breakdown feeds the pool of acetyl-CoA, the building block of polyketides and, namely, of amphidinol A (**1**). Therefore, inhibition of the glycolate/glyoxylate transformation was expected to arrest fast glycolate catabolism, avoiding scrambling of the labelling from the [1-^13^C]-glycolate precursor (Figure 5).

**Figure 6:**
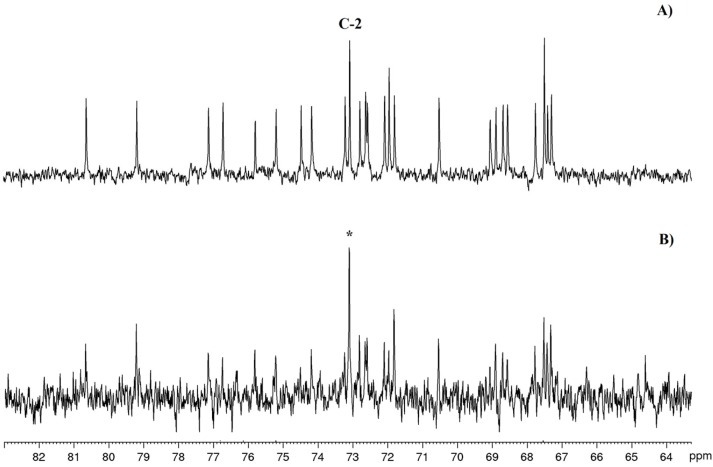
^13^C NMR spectra enlargements of **1**. (**A**) Natural abundance; (**B**) after feeding experiment with [1-^13^C] glycolate/SHAM. (*) indicates labelled carbon.

Following this line of reasoning, we decided to co-administer [1-^13^C]-glycolate and SHAM to cultures of *A. carterae*. Under the same conditions used for the other feeding experiments, extraction of microalgal cells gave amphidinol A (**1**) with a simplified enrichment pattern, as shown in Figure 4C. The carbon labelling appeared to be restricted to the incorporation of a single carbon, as suggested by M+1 and M+2 signal increases with respect to the mass spectrum of the unlabeled product. Accordingly, the ^13^C NMR spectrum showed remarkable incorporation only in the signal at 73.1 ppm assigned to C2 (Figure 6), as expected if [1-^13^C]-glycolate is used as the starter unit.

In conclusion, we have identified two new members of the amphidinol family, here named amphidinol A (**1**) and amphidinol B (**2**), from a dinoflagellate strain of *A. carterae* isolated from Lake Fusaro, a brackish lagoon near Naples (Italy). Compound **1** showed a mild antifungal activity. Biosynthetic studies on amphidinol A were addressed with stable labelled acetate and glycolate and proved, for the first time, that glycolate is the starter unit of PKS assembly leading to this class of metabolites. This finding completes the characterization of the biogenesis of these complex microalgal polyketides that show several points of skeleton rearrangements and carbon deletions. Behind the aim of this work, the results of biosynthetic experiments with [1-^13^C]-glycolate may have positive implications in the study of carbon metabolism in dinoflagellates.

## 3. Materials and Methods

### 3.1. General Experimental Procedures

Optical rotations were measured on a Jasco P2000 digital polarimeter (Jasco, Cremella, Italy). UV spectra were acquired on a Jasco V-650 spectrophotometer, NMR spectra were recorded on a Bruker Avance DRX 600 (Bruker, Milan, Italy) equipped with a cryoprobe operating at 600 MHz for protons. Chemical shift values are reported in ppm (δ) and referenced to internal signals of residual protons (CD_3_OD ^1^H δ3.34, ^13^C 49.0 ppm). High-resolution mass spectra were acquired on a Q-Exactive Hybrid Quadrupole-Orbitrap mass spectrometer (Thermo Scientific, Milan, Italy); HPLC analyses have been performed on a Jasco system (PU-2089 Plus-quaternary gradient pump equipped with a Jasco MD-2018 Plus photodiode array detector). [1-^13^C]-acetate, [2-^13^C]-acetate and [1,2-^13^C_2_]-acetate, [1-^13^C]-glycolate (all sodium salts), and salicylhydroxamic acid (SHAM) were obtained from Sigma Aldrich (Milan, Italy). Solvents were purchased from VWR (Milan, Italy) and were HPLC-grade.

### 3.2. Biological Material

*Amphidinium carterae* was isolated in June 2012 from a sample of *Dyctiota dichotoma* collected from Lake Fusaro, near Naples, Italy. Once transferred to the lab, kept in containers filled with seawater, the macroalgae *D. dichotoma* were shaken vigorously to enhance epiphyte detachment. Zooplankton and larger animals were removed by gentle filtering onto a 30 µm nylon mesh. Seawater subsamples were then transferred to Petri dishes. The dinoflagellate cells were isolated by capillary pipettes and initially grown in multiwell plates filled with K medium [33]. Species identification was performed by FEM2-Ambiente Srl (Milan, Italy) and the strain is available in our lab, coded as ICB-BCL0018.

*Amphidinium carterae* was cultured in K medium at 22.0 ± 0.5 °C, under a 14:10 h light:dark regime and at 100 µmol m^−2^ s^−1^. The cells were mass-cultivated in four sterile 1.8 L glass Fernbach bottles, each containing 1 L of culture. The initial cell density was around 8000 cells/mL. During the exponential phase, each subculture was halved, and refreshed with half a liter of K medium. In the stationary phase (final cell density: 230,000 cells/mL), the eight liters of culture were harvested in a swing-out centrifuge (Allegra 12-XR, Beckman Coulter, Milan, Italy), for 10 min at 4 °C at 2300× *g*. The cell pellet was stored at –80 °C until analysis.

### 3.3. Extraction and Isolation of Amphidinols **1** and **2**

The cell pellet of *A. carterae* (wet weight 1.9 g) was extracted with methanol (3 × 10 mL) by sonication and centrifuged to remove cell debris. The methanol phase was filtered through paper and concentrated under vacuum. The crude extract (180 mg) was fractionated on a Chromabond C-18 Hydra column (5 g of dry resin ) (Macherey-Nagel, Düren, Germany) by using the following stepwise elution protocol: A (100% H_2_O, 40 mL), B (25% MeOH, 40 mL), C (50% MeOH, 40 mL), D (75% MeOH, 40 mL), and E (100% MeOH, 80 mL). Fraction D (6.4 mg) was further purified on a RP-HPLC column (C18-Luna, Phenomenex, 5 µm 100 A, 250 × 10 mm) by a MeOH/H_2_O initial gradient from 65% to 80% of MeOH for 20 min, followed by a gradient to 100% MeOH for 15 min (flow 3 mL/min). UV absorbance at 210 nm was used for peak detection. Pure compound **1** (1.6 mg) was eluted at Rt = 23 min. Fraction C (2.5 mg) was purified by HPLC, as above, affording 1.0 mg of pure compound **2** (Rt = 8 min).

**Amphidinol A (1).** Pale yellow amorphous solid. [α]_25_^D^ +3 (*c* 0.14, MeOH); UV (MeOH) *λ*_max_ (log *ε*) 210 (4.75) nm; NMR see Table 1. HR-ESIMS *m*/*z* 1361.8532 [M+Na]^+^ (calcd for C_69_H_126_O_24_Na^+^, 1361.8531).

**Amphidinol B (2).** Pale yellow amorphous solid. [α]_25_^D^ +1 (*c* 0.16, MeOH); UV (MeOH) *λ*_max_ (log *ε*) 210 (4.30) nm; NMR Data see Table 1. HR-ESIMS [M-H]^−^ *m*/*z* 1417.8187 (calcd for C_69_H_125_SO_27_^−^1417.8134); [M-H+2Na]^+^ *m*/*z* 1463.7912 (calcd for C_69_H_125_ SO_27_ Na_2_^+^ 1463.7919).

### 3.4. Biosynthetic Experiments

#### 3.4.1. Control

The culture used as the control of the biosynthetic experiments was grown in a sterile 1.8 L glass Fernbach bottle, containing 1.5 L of culture medium with antibiotics (35 µg/mL of Penicillin G and 50 U/mL of streptomycin) added together with inoculation. The initial cell density was around 8000 cells/mL. Cells were harvested after three weeks in their stationary phase (with a final cell density of 250,000 cells/mL) by a swing-out centrifuge Allegra 12-XR (Beckman Coulter, Milan, Italy), for 10 min at 4 °C at 2300 *g*. The cell pellet (wet weight 400 mg) was stored at −80 °C before chemical analysis.

#### 3.4.2. Feeding Experiments with Labelled Acetate

##### Experiments with [1-^13^C]-Acetate

[1-^13^C]-acetate (0.6 mM) was added to culture medium (1.5 L of K medium in 5 × 1.8 L glass Fernbach bottles) together with antibiotics and inoculated as above. After three weeks, at the stationary phase, the cell density reached 200,000 cells/mL and the culture was harvested as above. The cell pellets (wet weight 1.9 g) was extracted with MeOH affording 110 mg of raw extract which was purified as previously described. Briefly, the extract was loaded onto a Chromabond C18 Hydra cartridge (6 mL/500 mg) and eluted as indicated above (8 mL each step). Further purification by RP-HPLC of the fraction D (7.2 mg) led to the isolation of 2 mg of labelled amphidinol A (**1**).

##### Experiments with [2-^13^C]-Acetate

[2-^13^C]-acetate (0.6 mM) was added to culture medium (1.5 L of K medium in 1.8 L glass Fernbach bottle) together with antibiotics and inoculated as above. After 21 days, at the stationary phase cell density reached 71,000 cells/mL and the culture was harvested as above. The cell pellets (wet weight 270 mg) was extracted with MeOH affording 15.5 mg of raw extract and purified as previously described using a cartridge of Chromabond C18 Hydra (6 mL/500 mg) and 8 ml of solvent for the stepwise elution. Further purification by RP-HPLC of the fraction D eluted with 75% MeOH (1.3 mg) led to the isolation of <0.1 mg of labeled polyketide **1**.

##### Experiments with [1,2-^13^C_2_]-Acetate

[1,2-^13^C_2_]-acetate (0.6 mM) was added to culture medium (1.5 L of K medium in 5 × 1.8 L glass Fernbach bottles) together with antibiotics and inoculated as above. After 21 days, at the stationary phase, the cell density reached 84,000 cells/mL and the culture was harvested as above. The cell pellet (wet weight 270 mg) was extracted with MeOH, affording 19.5 mg of raw extract and purified as previously described using a cartridge of Chromabond C18 Hydra (6 mL/500 mg) and 8 mL of solvent for the stepwise elution. Further purification by RP-HPLC of the fraction eluted with 75% MeOH (1.6 mg) led to the isolation of 0.2 mg of labeled product **1**.

#### 3.4.3. Feeding Experiments with Labelled Glycolate

##### Experiments with [1-^13^C]-Glycolate

Two sterile 1.8 L glass Fernbach bottles, containing 1.5 L of inoculated culture treated with antibiotics were grown as described above with 0.42 mM [1-^13^C]-glycolate. After 21 days, when the final cell density was around 130,000 cells/mL, the culture was centrifuged and the humid pellet (834 mg) was extracted with MeOH affording 82.7 mg of extract. Two step fractionation by C18 Hydra resin (2.3 g) followed by RP-HPLC, led to 1 mg of pure labelled product **1**.

##### Experiments with [1-^13^C]-Glycolate/Salicylhydroxamic Acid (SHAM)

[1-^13^C]-glycolate (0.42 mM), SHAM (0.5 mM), and antibiotics (35 µg/mL Penicillin G and 50 U/mL streptomycin) were added simultaneously with inoculation. The cell culture (150 mL, 8000 cells/mL) was harvested after three weeks (356,000 cells/mL). A second algal culture control with 0.5 mM of inhibitor and without labelled glycolate was grown under the same conditions.

The cell pellet (119 mg) from the experiment with labeled glycolate and SHAM was extracted with MeOH to obtain 11.9 mg of raw material. By fractionation with a C18 Hydra Chromabond cartridge (3 mL/200 mg) with the usual elution gradient (5 mL for each solvent) two enriched fractions C (0.5 mg) and D (0.7 mg) were obtained. Further purification by HPLC, as described above, led to the isolation of labelled amphidinol A (**1**, 0.1 mg) and B (**2**, <0.1 mg). The polyketide profile in the control culture with SHAM was unaffected by the presence of the inhibitor.

### 3.5. Antifungal Test

Antifungal activity was tested against *Candida albicans* (ATCC90028) with the broth microdilution liquid growth inhibition method in sterile 96-well plates. The final fungal cell concentration was 1 × 10^4^ CFU/mL. Plates were incubated at 37 °C for 48 h. Results have been reported as MIC (minimum inhibitory concentration) in µg/mL.

## Figures and Tables

**Table 1:** NMR data (600 MHz) of amphidinol A (**1**) and B (**2**).

Position	Type	1		2		Position	Type	1		2	
		CD_3_OD/C_5_N_5_D (2:1)		CD_3_OD				CD_3_OD/C_5_N_5_D (2:1)		CD_3_OD	
		δ_C_	δ_H_, mult, *J* (Hz)	Type	δ_H_, mult, *J* (Hz)			δ_C_	δ_H_, mult, *J* (Hz)	δ_C_	δ_H_, mult, *J* (Hz)
**1**	CH_2_	67.6	3.55, m; 3.59, m	67.1	3.44, dd, 11.0, 6.3; 3.51, m	**33**	CH	72.7	3.87, dd, 9.0, 2.0	72.4	3.70, m
**2**	CH	73.1	3.71, m	73.0	3.61, m	**34**	CH	79.2	4.26, dd, 9.5, 2.0	79.1	3.99 ^d^, m
**3**	CH_2_	34.7	1.47, m; 1.57, m	34.2	1.41, m	**35**	CH	69.0	4.34, m	67.2	4.07, m
**4**	CH_2_	26.8	1.40, m; 1.46, m	26.6	1.43,m; 1.52, m	**36**	CH	67.2	4.16, m	67.2	4.00, m
**5**	CH_2_	26.9	1.47, m; 1.55, m	25.8	1.48, m	**37**	CH_2_	30.7	1.98, dt, 12.0, 3.6; 2.05, q, 12.0	30.2	1.82, m
**6**	CH_2_	38.6	1.46, m; 1.52, m	35.2	1.50, m; 1.70, m	**38**	CH	75.8	3.65 ^b^, m	75.5	3.55 ^c^, m
**7**	CH	71.8	3.58, m	80.3	4.39 ^a^, m	**39**	CH	74.5	3.75, m	74.0	3.64, m
**8**	CH_2_	38.6	1.46, m; 1.52, m	35.3	1.50, m; 1.70, m	**40**	CH_2_	32.6	1.73, m; 2.12, m	32.1	1.60, m; 2.0, m
**9**	CH_2_	22.9	1.45, m; 1.70, m	21.9	1.49, m; 1.60, m	**41**	CH_2_	27.9	2.27, m	27.7	2.15, m; 2.45, m
**10**	CH_2_	38.6	1.46, m; 1.52, m	38.4	1.48, m; 1.55, m	**42**	C	152.0	-	151.3	-
**11**	CH	68.6	4.18, m	68.6	4.09, m	**43**	CH	76.8	4.43, d, 8.6	76.4	4.22, d, 8.8
**12, 14**	CH_2_	51.9	2.69, m	51.6	2.65, m	**44**	CH	75.3	3.54, m	75.1	3.38, m
**13**	C	211.2	-	211.6	-	**45**	CH	70.6	4.25, dt, 12.0, 2.0	70.4	4.07, m
**15**	CH	68.9	4.13, m	68.8	4.07, m	**46**	CH_2_	31.9	1.68, m; 2.36, q, 12.0	31.5	1.60, m; 2.12, m
**16**	CH_2_	35.7	1.49, m; 1.59, m	35.5	1.40, m; 1.50, m	**47**	CH	67.3	4.21, dd, 10.0, 3.5	69.2	4.07 ^e^, m
**17**	CH_2_	33.1	1.14, m;1.72, m	33.0	1.15, m; 1.62, m	**48**	CH	68.6	4.33, m	68.6	4.07 ^e^, m
**18**	CH_2_	30.6	1.76, m	30.2	1.70, m	**49**	CH	80.7	4.02, dd, 10.0, 1.5	80.4	3.78 m
**19**	CH_2_	41.3	1.47, m; 1.55, m	41.8	1.40, m; 1.50, m	**50**	CH	72.0	4.22, m	71.9	3.99 ^d^, m
**20**	CH	73.3	3.65 ^b^, m	73.3	3.55 ^c^, m	**51**	CH	74.2	4.62, dd, 10.0, 1.5	74.1	4.39 ^a^, m
**21**	CH	72.6	3.69, m	72.6	3.5, m	**52**	CH	129.1	5.79, dd, 15.5, 7.0	128.1	5.63, dd, 15.0, 7.5
**22**	CH_2_	38.7	1.57, m; 1.88, m	38.3	1.40, m; 1.50, m	**53**	CH	135.3	5.85, dt, 15.5, 5.7	135.6	5.82, dt, 15.0, 6.5
**23**	CH_2_	31.3	2.40, m	31.0	2.18, m	**54**	CH_2_	34.6	1.99, m	33.2	2.10, m
**24**	CH	77.2	3.57, m	77.2	3.39, m	**55**	CH_2_	29.9	1.30, m	30.8	1.44, m
**25**	CH	72.8	3.86, m	72.5	3.71, m	**56–62**	CH_2_	30.1–30.8	1.21–1.30, m	30.0-30.6	1.33–1.35, m
**26**	CH_2_	41.9	1.65, m; 2.16, m	41.6	1.56, 2.02	**63**	CH_2_	34.6	1.97, m	33.4	2.08, m
**27**	CH	71.8	4.00, m	71.4	3.90, m	**64**	CH	140.1	5.77, m	135.8	5.81, m
**28**	CH_2_	36.9	1.67, m	36.7	1.63, m; 1.71, m	**65**	CH_2_	115.0	4.91, brd, 11.0;	114.3	4.94, brd, 10.7;
**27**	CH	71.8	4.00, m	71.4	3.90, m	**64**	CH	140.1	4.97, brd, 17.0	135.8	5.01 ^f^
**29**	CH_2_	36.4	2.12, m; 2.27, m	36.4	2.16, m; 2.24, m	**66**	CH_3_	21.3	0.91, d, 7.0	20.8	0.99, d, 6.9
**30**	C	138.4	-	139.2	-	**67**	CH_3_	14.2	1.05, d, 6.5	13.7	0.97, d, 7.0
**31**	CH	126.1	5.66, d, 9.0	126.0	5.52, d, 8.5	**68**	CH_3_	17.4	1.75, s	17.0	1.78, s
**32**	CH	67.5	4.76, dd, 9.0, 2.0	67.6	4.58, dd, 8.5, 1.5	**69**	CH_2_	113.0	5.03, s; 5.17, s	112.7	5.01 ^f^, s; 5.10, s

^a–f^ Overlapping signals with the same superscript letter.

## Supplementary Materials

The following are available online at www.mdpi.com/1660-3397/15/6/157/s1, Figures S1–S20: NMR data, Figures S21–S23: ESIMS data.
