# Supplementary material for: The Missing Piece in Biosynthesis of Amphidinols: First Evidence of Glycolate as a Starter Unit in New Polyketides from Amphidinium carterae
Source: Mar Drugs. 2017 May 31;15(6):157. doi: 10.3390/md15060157 (PMC5484107; doi:10.3390/md15060157)
Supplement: Supplementary file 1 [file marinedrugs-15-00157-s001.pdf]

Supplementary Materials: The Missing Piece in Biosynthesis of Amphidinols: First Evidence of Glycolate as a Starter Unit in New Polyketides from *Amphidinium carterae*

Adele Cutignano, Genoveffa Nuzzo, Angela Sardo and Angelo Fontana

Figure S1. ^{1}H NMR spectrum (600 MHz, CD_3OD/C_5D_5N 2:1) of **1**.
Figure S2. ^{13}C NMR spectrum (600 MHz, CD_3OD/C_5D_5N 2:1) of **1**.
Figure S3. ^{13}C Jmod spectrum (600 MHz, CD_3OD/C_5D_5N 2:1) of **1**.
Figure S4. ^{1}H-^{1}H COSY spectrum (600 MHz, CD_3OD/C_5D_5N 2:1) of **1**.
Figure S5. TOCSY spectrum (600 MHz, CD_3OD/C_5D_5N 2:1) of **1**.
Figure S6. edHSQC spectrum (600 MHz, CD_3OD/C_5D_5N 2:1) of **1**.
Figure S7. HMBC (J = 7 Hz) spectrum (600 MHz, CD_3OD/C_5D_5N 2:1) of **1**.
Figure S8. NOESY (600 MHz, CD_3OD/C_5D_5N 2:1) of **1**.
Figure S9. ^{1}H NMR spectrum (600 MHz, CD_3OD) of **2**.
Figure S10. ^{13}C Jmod spectrum (600 MHz, CD_3OD) of **2**.
Figure S11. ^{1}H-^{1}H COSY spectrum (600 MHz, CD_3OD) of **2**.
Figure S12. TOCSY spectrum (600 MHz, CD_3OD) of **2**.
Figure S13. edHSQC spectrum (600 MHz, CD_3OD) of **2**.
Figure S14. HMBC (J = 7 Hz) spectrum in CD3OD (600 MHz) of **2**.
Figure S15. ^{13}C NMR spectrum (600 MHz, CD_3OD/C_5D_5N 2:1) of **1** from the experiment with [1-^{13}C]-acetate.
Figure S16. ^{13}C NMR spectrum (600 MHz, CD_3OD/C_5D_5N 2:1) of **1** from the experiment with [2-^{13}C]-acetate.
Figure S17. ^{13}C NMR spectrum (600 MHz, CD_3OD/C_5D_5N 2:1) of **1** from the experiment with [1,2-$^{13}C_2$]-acetate.
Figure S18. ^{13}C NMR spectrum (600 MHz, CD_3OD/C_5D_5N 2:1) of **1** from the experiment with [1-^{13}C]-glycolate.
Figure S19. ^{13}C NMR spectrum (600 MHz, CD_3OD/C_5D_5N 2:1) of **1** from the experiment with [1-^{13}C]-glycolate/SHAM.
Figure S20. ^{13}C NMR spectrum (600 MHz, CD_3OD) of **2** from the experiment with [1-^{13}C]-glycolate/SHAM.
Figure S21. ESI$^+$ MS/MS of **1** on molecular ion sodium adduct $[M+Na]^+$ at m/z 1361.8.
Figure S22. ESI$^-$ MS/MS of **2** on molecular ion $[M-H]^-$ at m/z 1417.8.
Figure S23. ESI$^+$ MS/MS of **2** on molecular ion sodium adduct $[M-H+2Na]^+$ at m/z 1463.8.

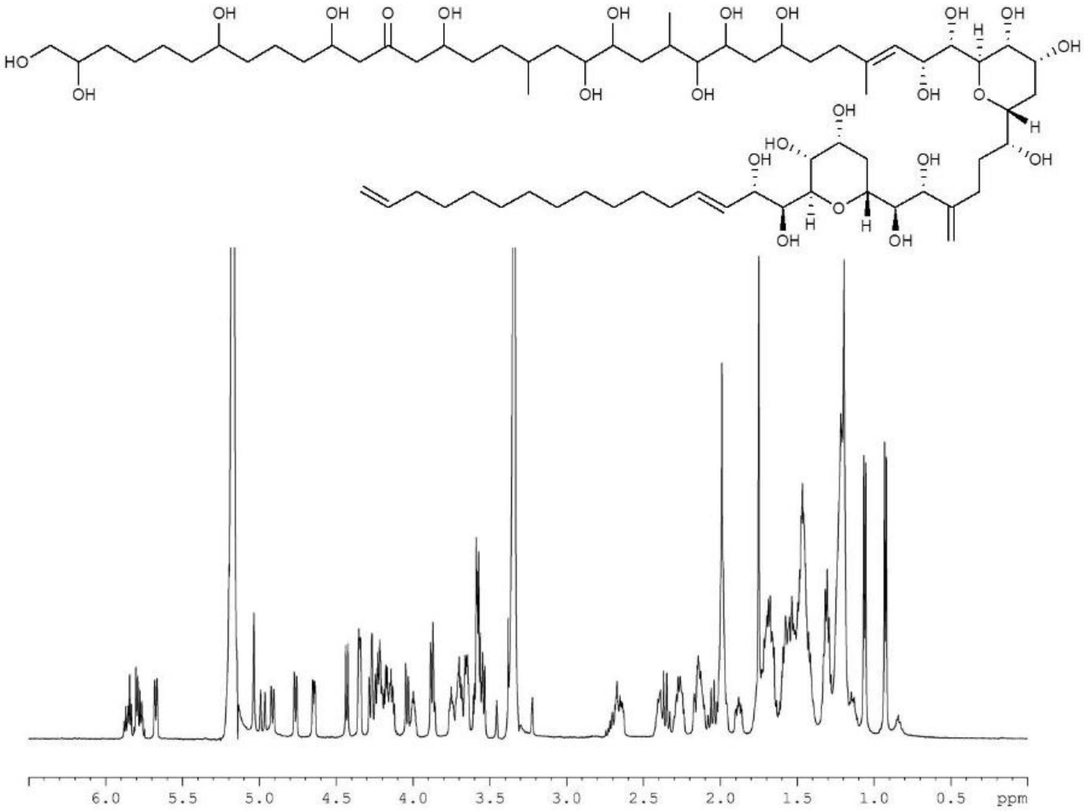

Figure S1. ^{1}H NMR spectrum (600 MHz, CD_3OD/C_5D_5N 2:1) of **1**.

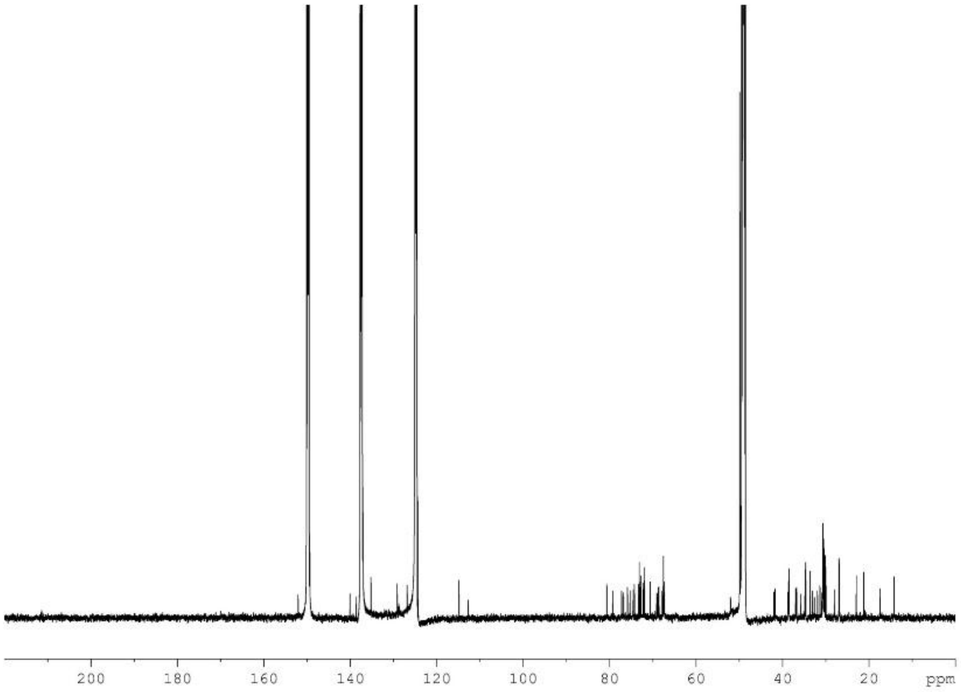

Figure S2. ^{13}C NMR spectrum (600 MHz, CD_3OD/C_5D_5N 2:1) of **1**.

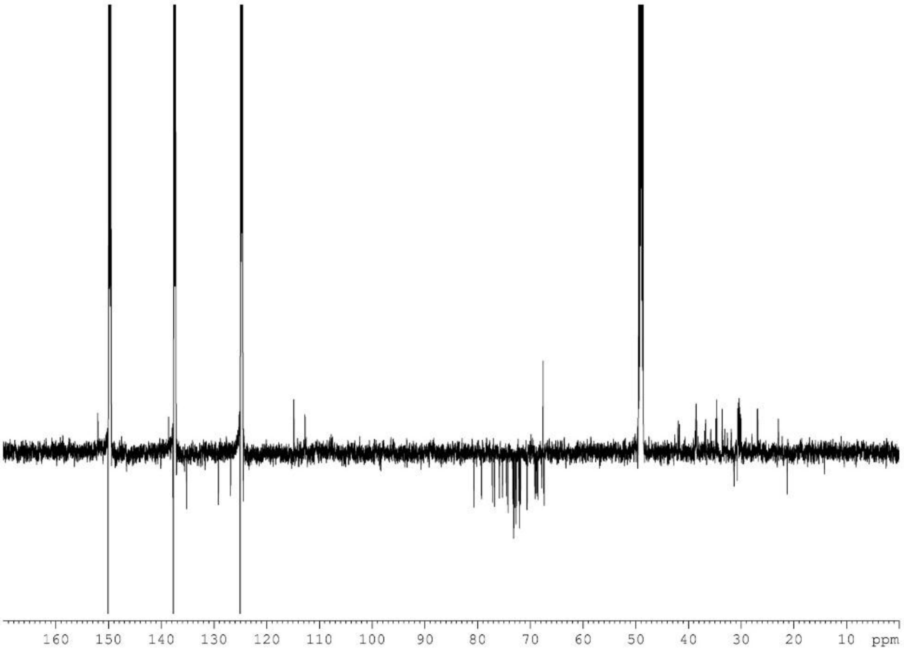

Figure S3. [13]C Jmod spectrum (600 MHz, CD_3OD/C_5D_5N 2:1) of **1**.

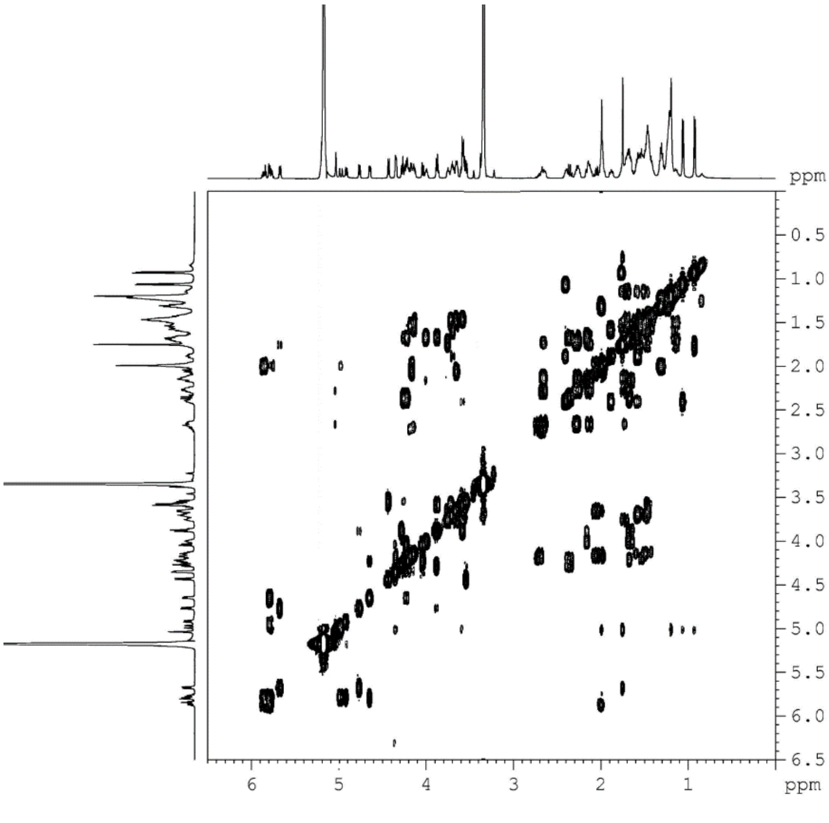

Figure S4. ^{1}H-^{1}H COSY spectrum (600 MHz, CD_3OD/C_5D_5N 2:1) of **1**.

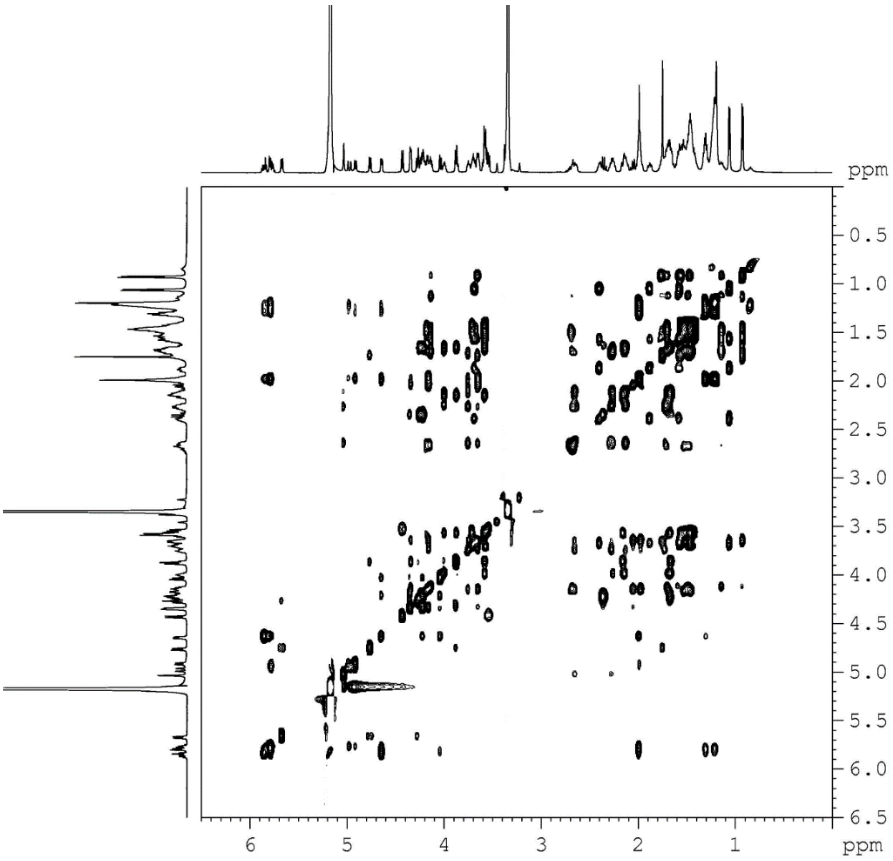

Figure S5. TOCSY spectrum (600 MHz, CD_3OD/C_5D_5N 2:1) of **1**.

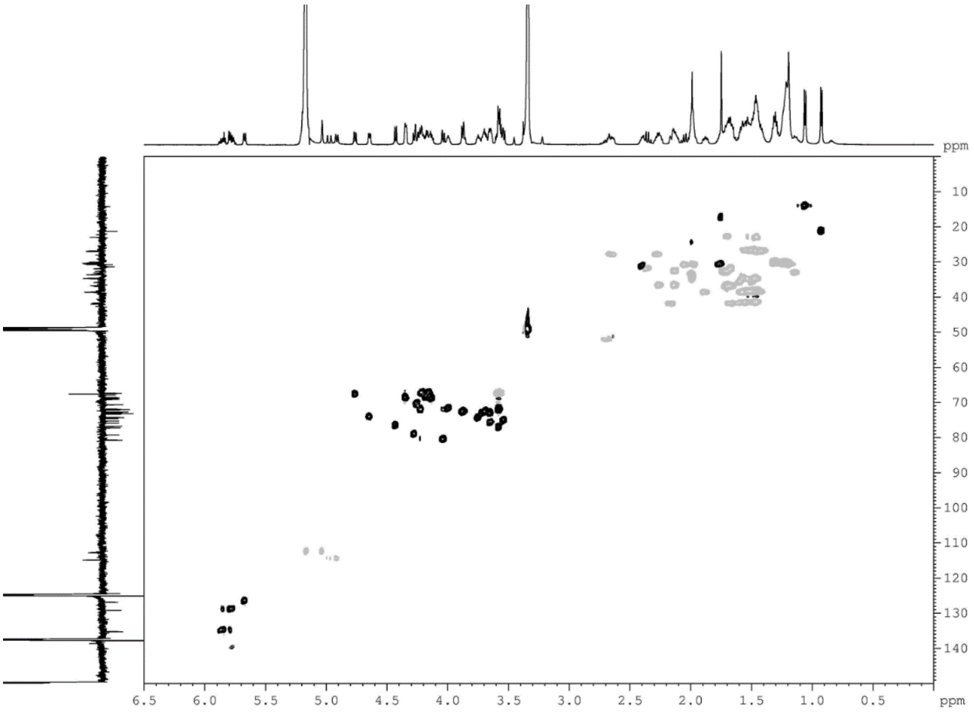

Figure S6. edHSQC spectrum (600 MHz, CD_3OD/C_5D_5N 2:1) of **1**.

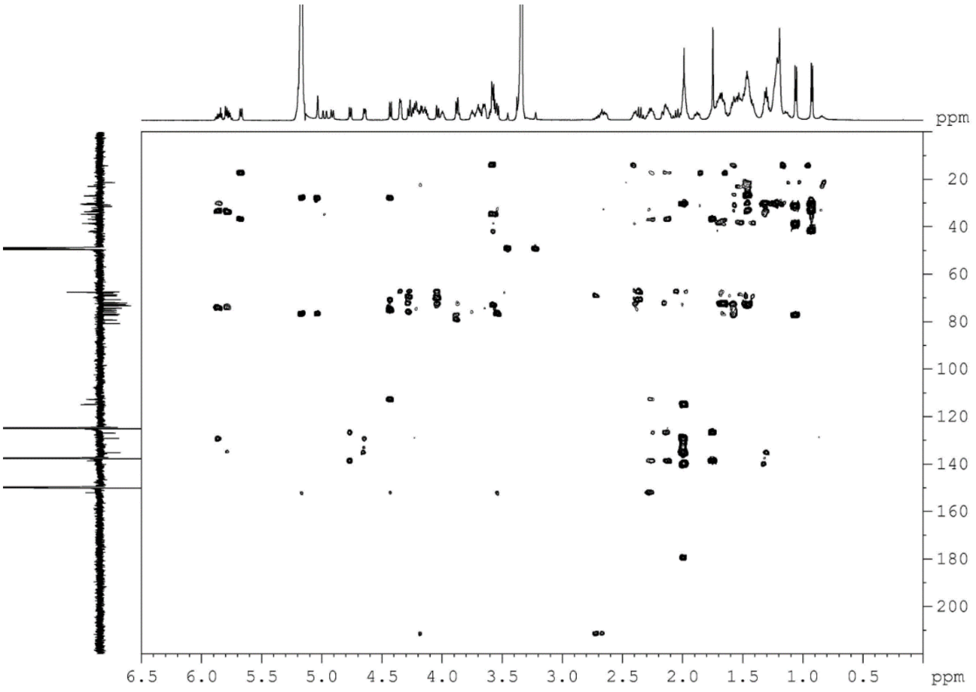

Figure S7. HMBC (J = 7 Hz) spectrum (600 MHz, CD_3OD/C_5D_5N 2:1) of **1**.

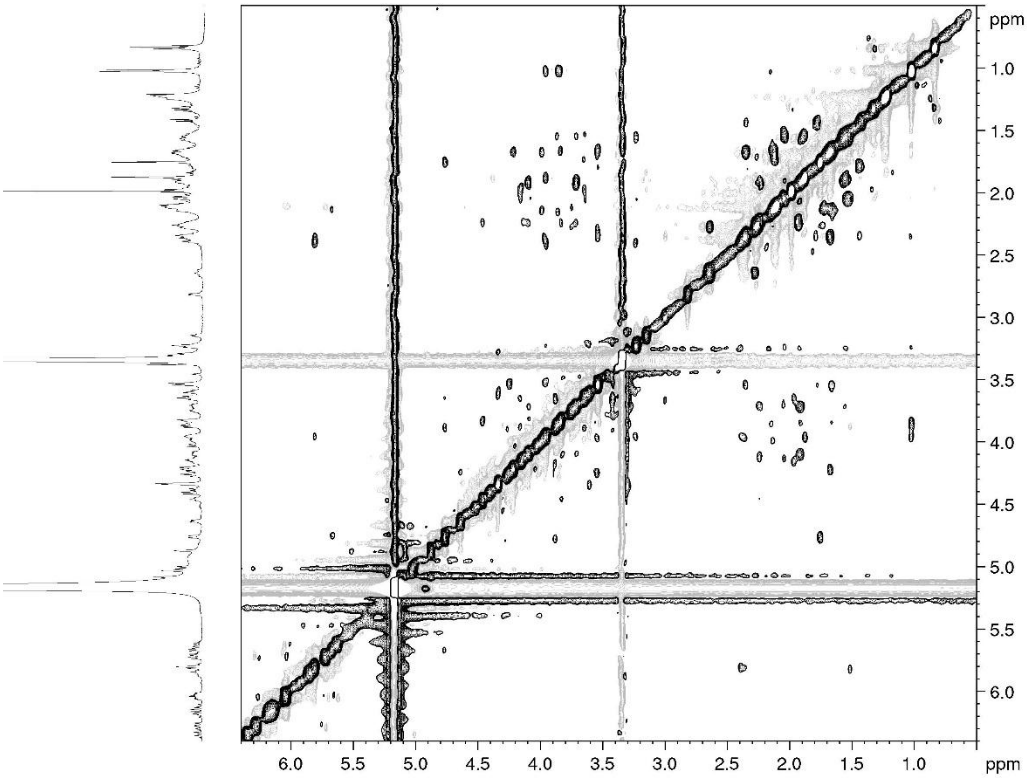

Figure S8. NOESY (600 MHz, CD_3OD/C_5D_5N 2:1) of **1**.

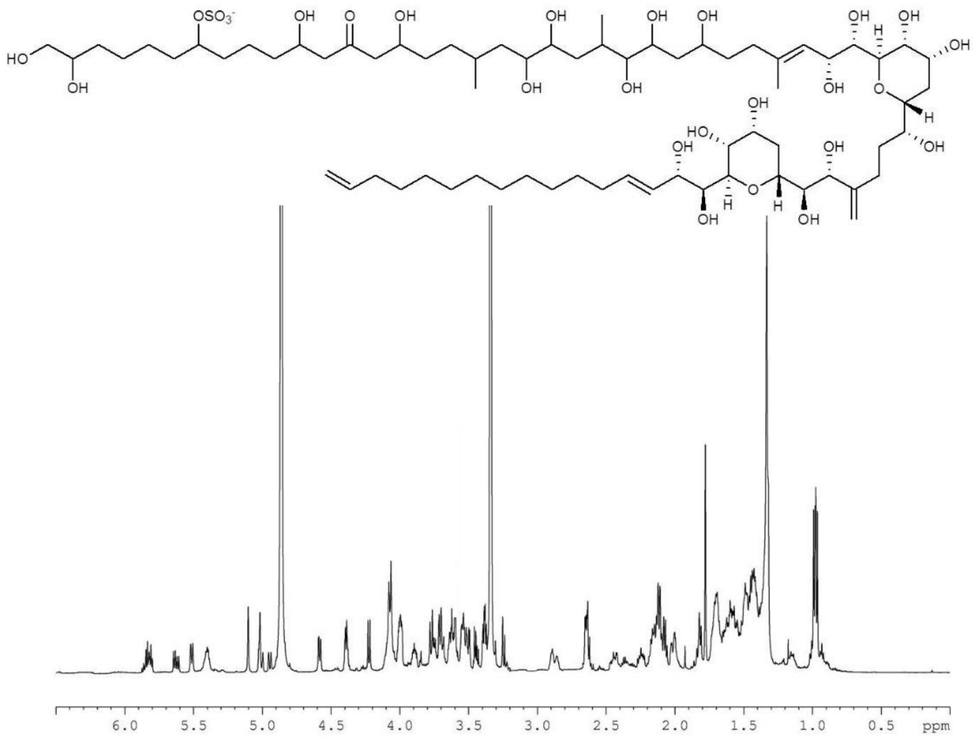

Figure S9. ^{1}H NMR spectrum (600 MHz, CD_3OD) of **2**.

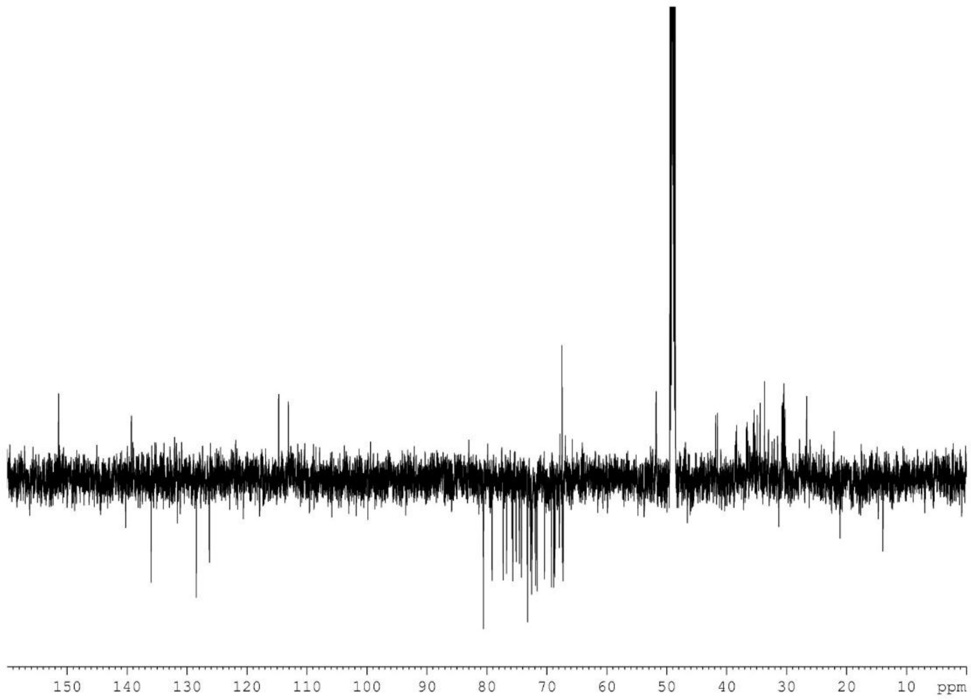

Figure S10. [13]C Jmod spectrum (600 MHz, CD_3OD) of **2**.

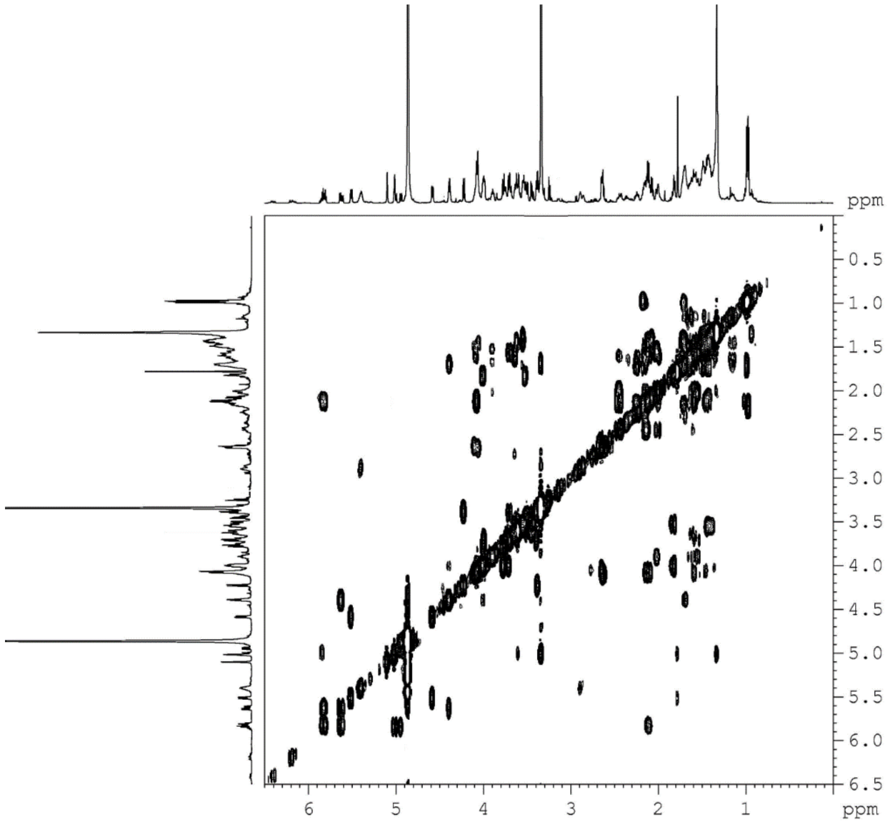

Figure S11. ^{1}H-^{1}H COSY spectrum (600 MHz, CD_3OD) of **2**.

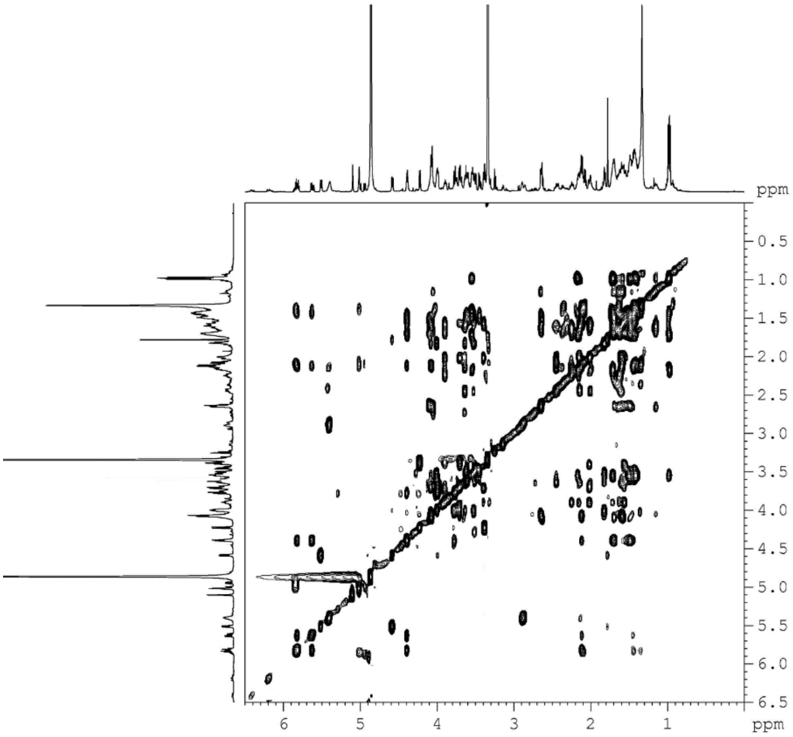

Figure S12. TOCSY spectrum (600 MHz, CD_3OD) of **2**.

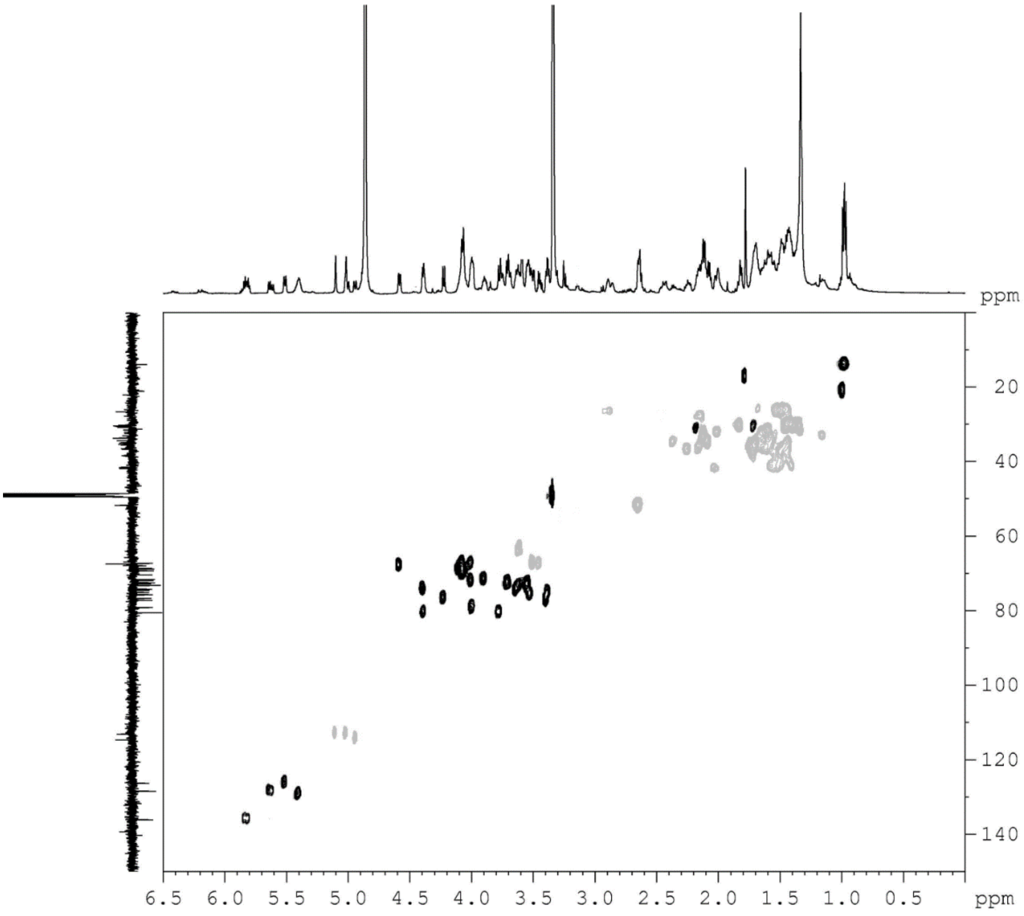

Figure S13. edHSQC spectrum (600 MHz, CD_3OD) of **2**.

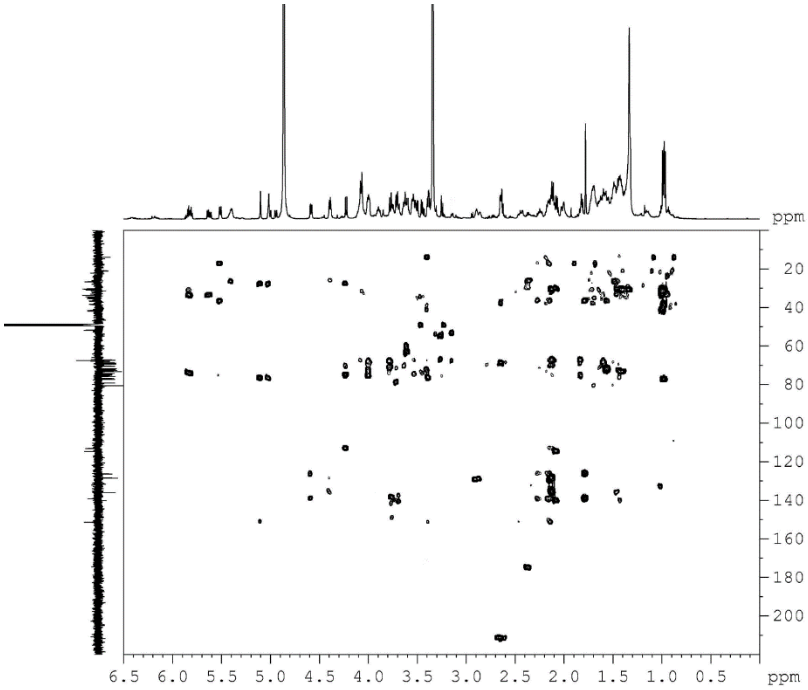

Figure S14. HMBC (*J* = 7 Hz) spectrum in CD_3OD (600 MHz) of **2**.

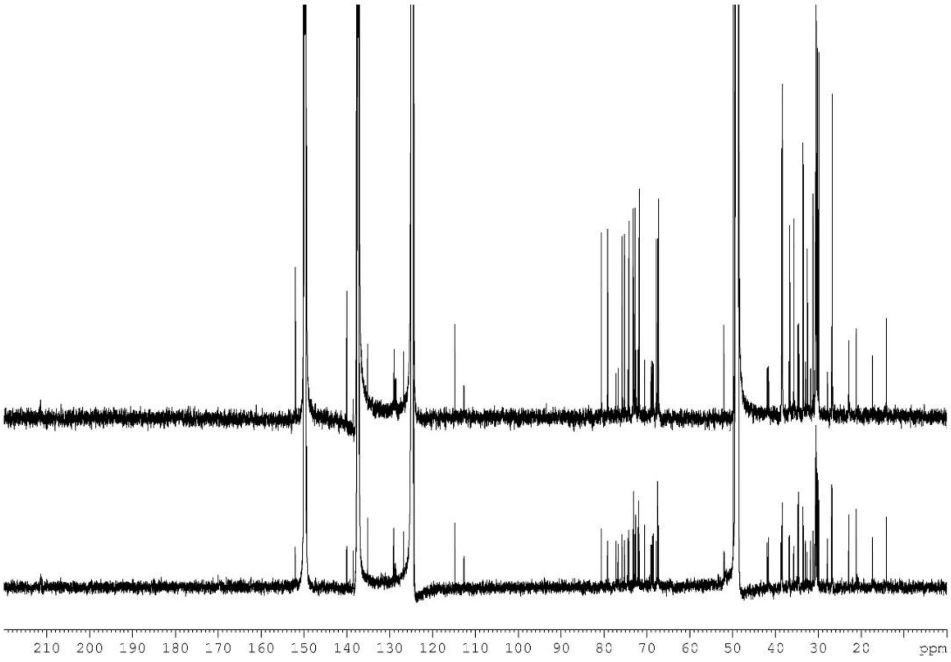

Figure S15. ^{13}C NMR spectrum (600 MHz, CD_3OD/C_5D_5N 2:1) of **1** from the experiment with [1-^{13}C]-acetate (upper) in comparison with the natural compound (lower).

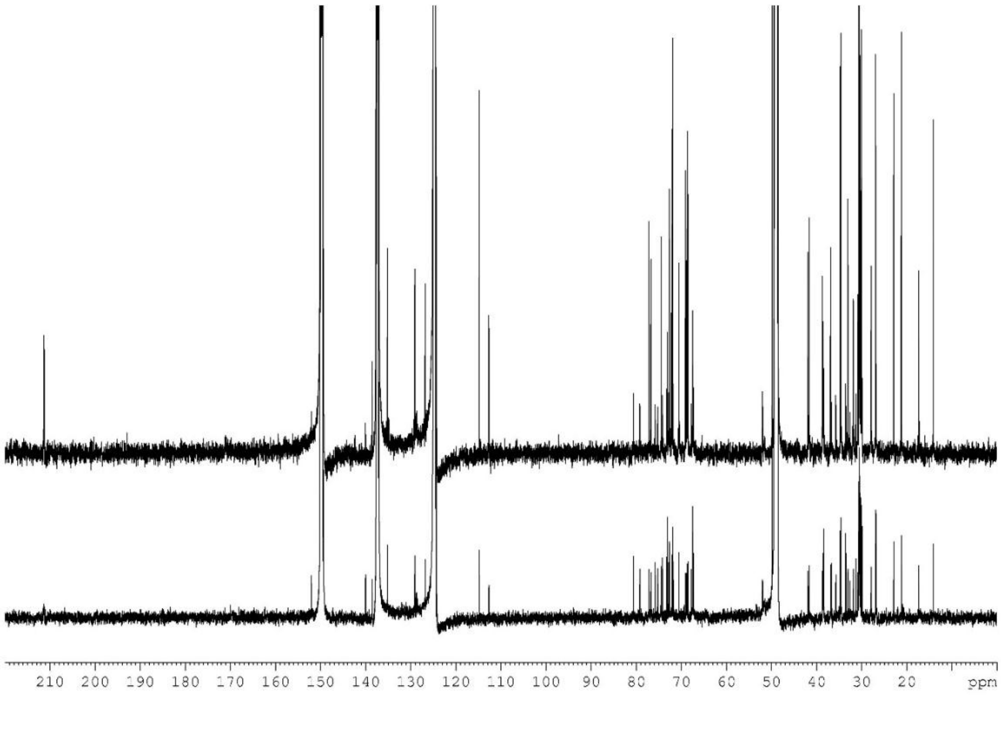

Figure S16. ^{13}C NMR spectrum (600 MHz, CD_3OD/C_5D_5N 2:1) of **1** from the experiment with [2-^{13}C]-acetate (upper) in comparison with the natural compound (lower).

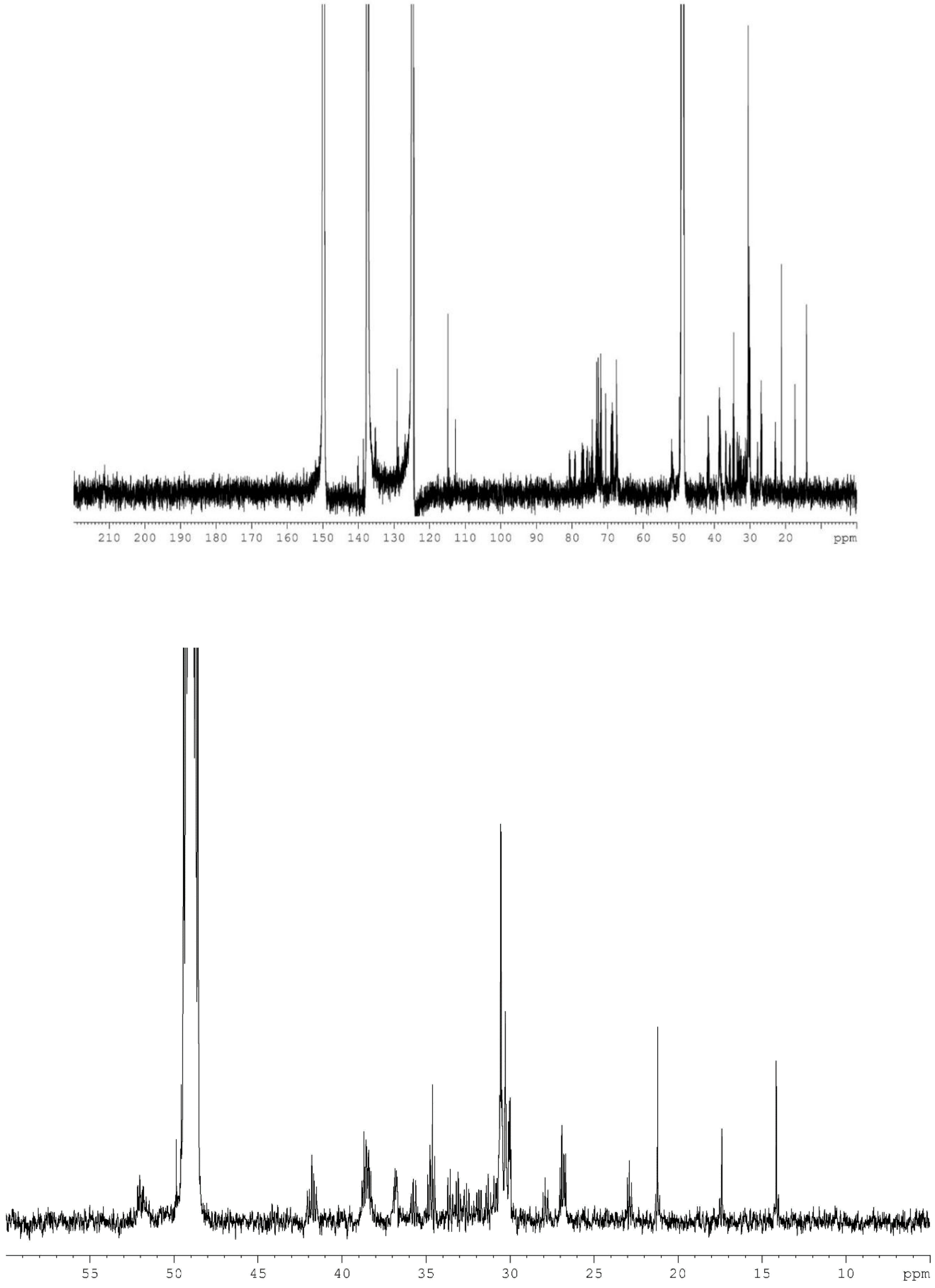

Figure S17. Two enlargments of ^{13}C NMR spectrum (600 MHz, CD_3OD/C_5D_5N 2:1) of **1** from the experiment with [1,2-$^{13}C_2$]-acetate.

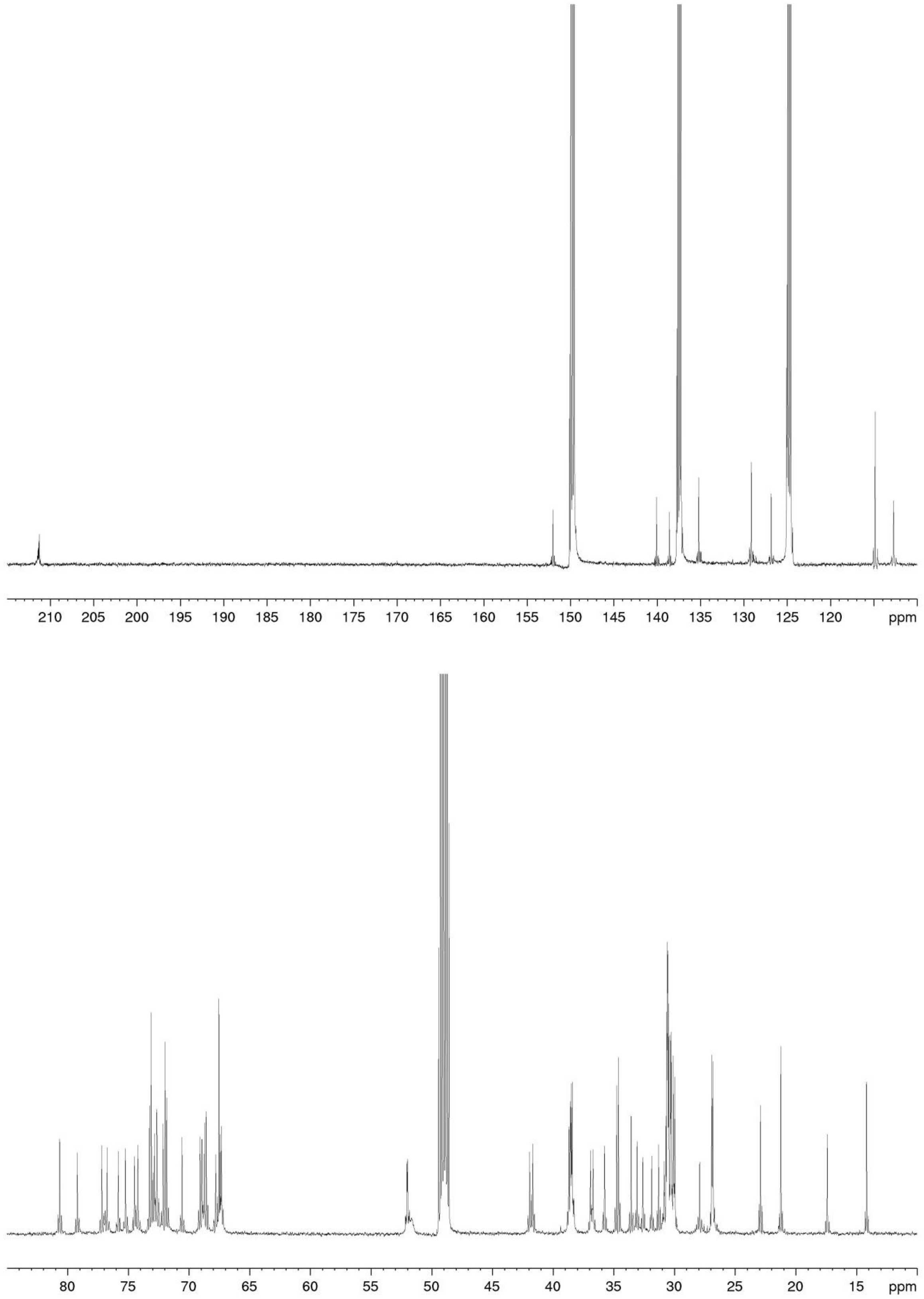

Figure S18. Two enlargments of ^{13}C NMR spectrum (600 MHz, CD_3OD/C_5D_5N 2:1) of **1** from the experiment with [1-^{13}C]-glycolate.

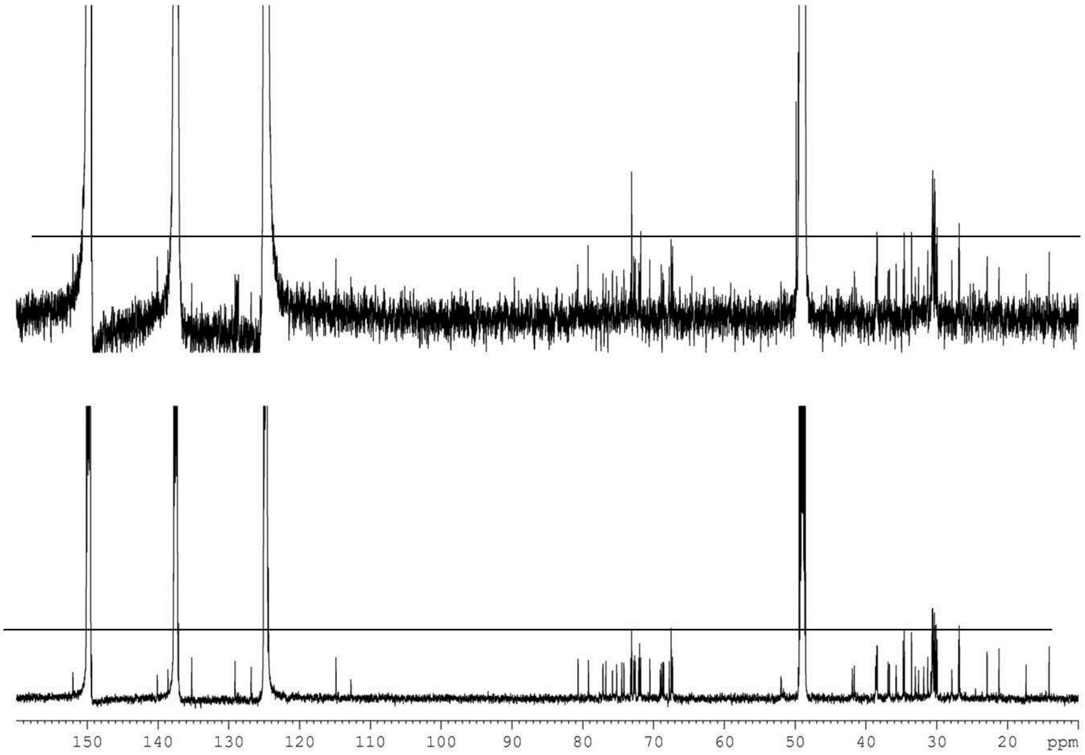

Figure S19. ^{13}C NMR spectrum (600 MHz, CD_3OD/C_5D_5N 2:1) of **1** from the experiment with [1-^{13}C]-glycolate/SHAM (upper) in comparison with the natural compound (lower).

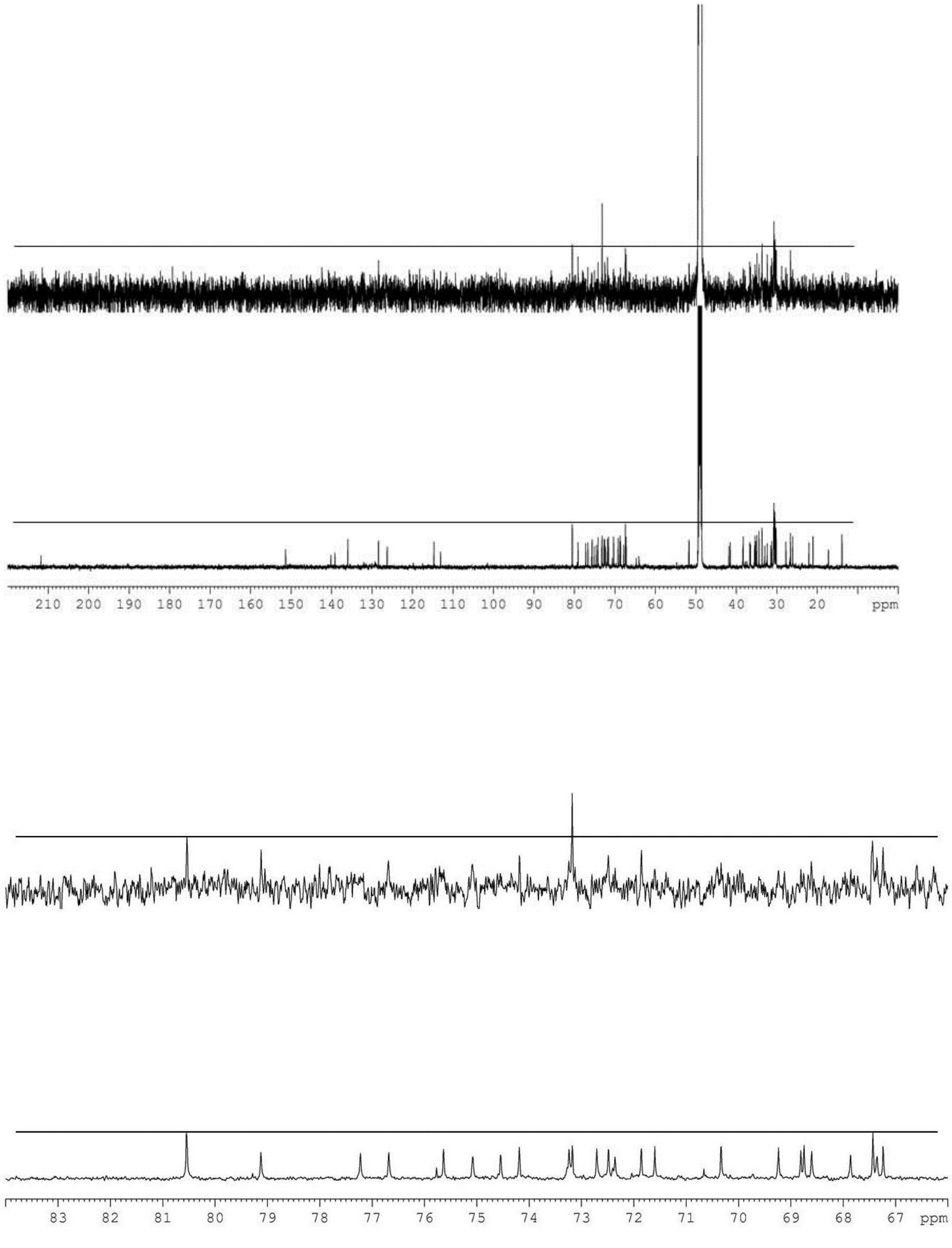

Figure S20. Two enlargements of ^{13}C NMR spectrum (600 MHz, CD_3OD) of **2** from the experiment with [1-^{13}C]-glycolate/SHAM (upper) in comparison with the natural compound (lower).

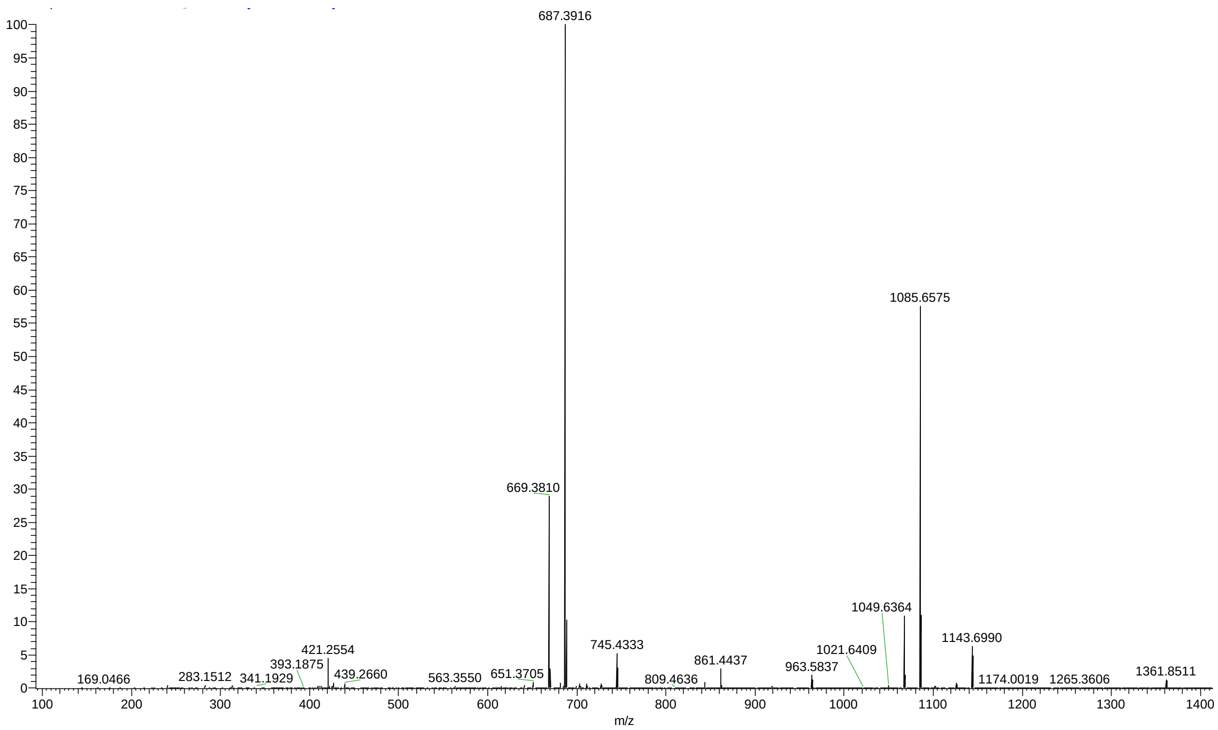

Figure S21. ESI+ MS/MS of **1** on molecular ion sodium adduct $[M+Na]^+$ at *m*/*z* 1361.8.

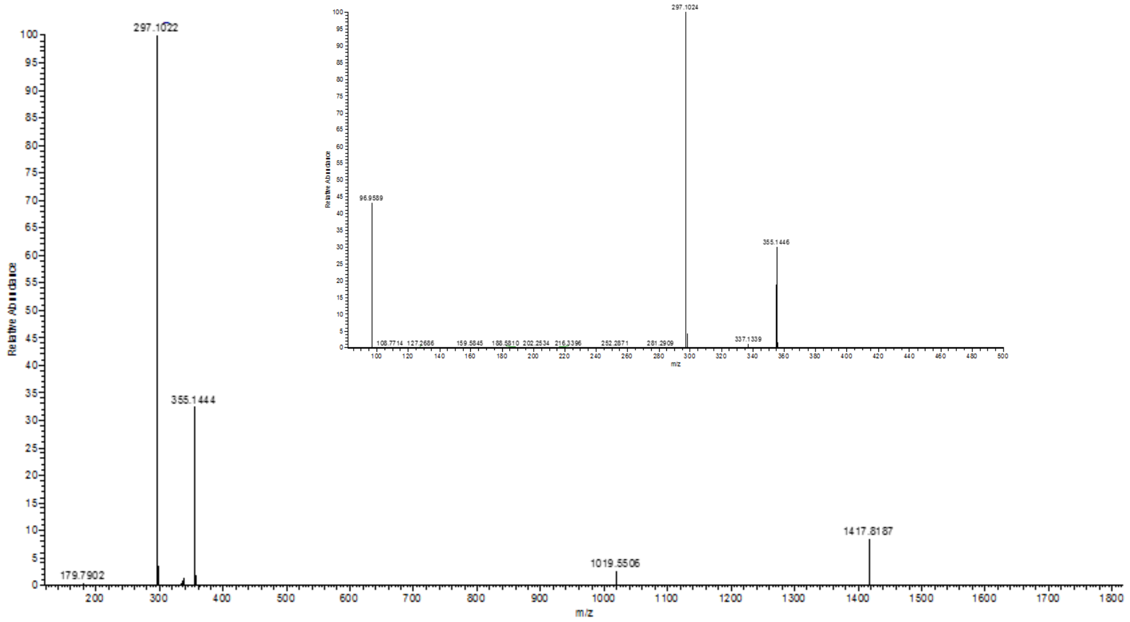

Figure S22. ESI- MS/MS of **2** on molecular ion $[M-H]^-$ at *m*/*z* 1417.8.

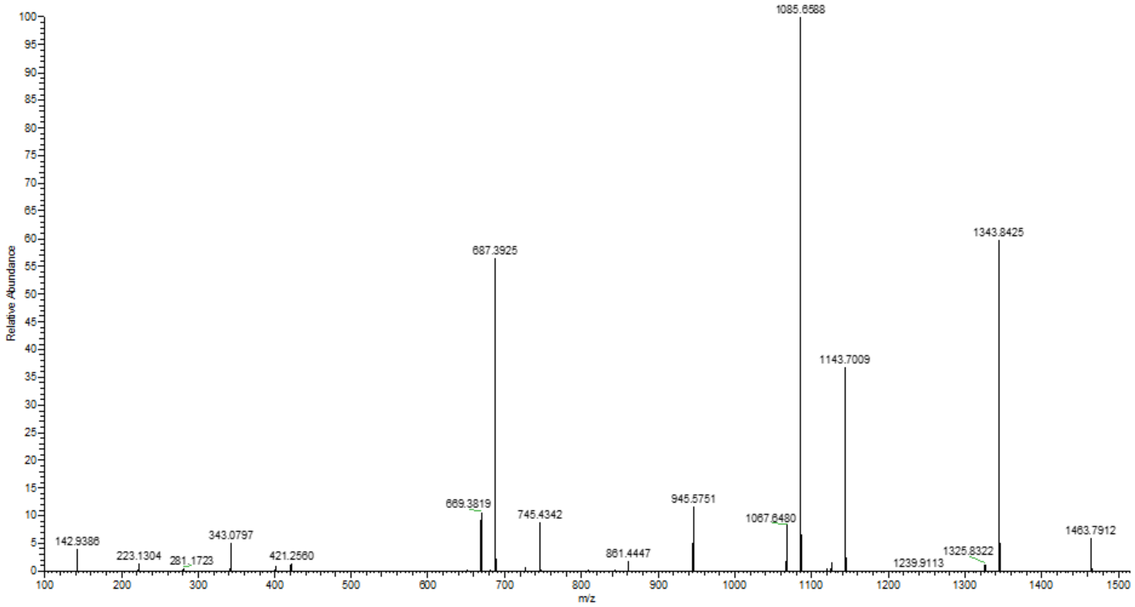

Figure S23. ESI+ MS/MS of **2** on molecular ion sodium adduct [M-H+2Na]+ at *m*/*z* 1463.8.
